# The Medical Institutions of the Presidency Town of Madras

**Published:** 1900-09-22

**Authors:** 


### THE MEDICAL INSTITUTIONS OF THE PRESIDENCY TOWN OF MADRAS.

One of the marked features of the report for the year 1898, and the triennial period ending therewith, is the steady increase in the number of women who seek accouchement at the hospitals. In the Government Maternity Hospital the rise has been from 1,117 patients in 1896 to 1,256 in 1898; in the Victoria Caste and Gosha Hospital, in charge of Dr. Florence Wells, from 78 to 141; and in Rajah Sir Ramaswami Mudelliar's Lying-in Hospital, from 507 to 526. Such an increase is particularly satisfactory, because there was throughout most of the institutions a considerable decrease in the total of patients under treatment during 1898. This was most apparent at the General and Ophthalmic Hospitals, which are usually well patronised by patients from the mofussil, and at the Royapettah Hospital, where Hindus and Mussulmans constitute the majority of those seeking relief. The cause is supposed to have been fear of being segregated on account of the prevalence of plague, and also the scarcity which prevailed during the year. The latter prevented the poorer eyots and others coming from such long distances owing to lack of funds. The general health of the population of Madras during 1898 was decidedly below par, small-pox prevailing in the early part of the year, and cholera in the months of July and September. Moreover, the number of cases of leprosy in the hospitals increased, not because the disease obtained a greater hold upon the people, but because the victims more frequently sought admission into hospital for the sake of board and lodging as well as for care and treatment. The Government Leper Hospital was unable to meet all the applications for admission. Some interesting details are given by the superintendent of this hospital as to the origin and treatment of leprosy. He draws attention to the fact that attempts to cultivate the bacillus of leprosy on artificial media, or to communicate leprosy to animals, have hitherto failed, although Campana, of Rome, claims to have succeeded in the former object with cultures from cases in active progress, and ascribes the failure of other observers to the fact that they used material from advanced cases in which the bacilli have degenerated. As a palliative remedy, experience shows that chalmugra oil administered in doses of from 5 to 60 minims twice daily, either alone or combined with an equal quantity of jungle almond oil, seems the most successful. Perchloride of mercury given subcutaneously in the early or eruptive stage has also been attended with remarkable benefit, and there are several advocates of the serum treatment, but the superintendent sadly admits that the hospital is as yet unequipped with any of the modern instruments or apparatus requisite for the pursuit of experimental work, "hence the large field it offers for such enquiries is still a fallow one, and its wealth of clinical materials remains unutilised." The professional report from the General Hospital shows a decrease of 388 patients in 1898, as compared with the previous year. Amongst the cases which were attended to during the twelve months there was an interesting example of beri-beri, the only one in eight years, and six of tetanus, of which three recovered. These were all treated with large (20 grains) doses of antipyrine. In the last week or fortnight towards the termination of every case, the temperature charts showed an irregular elevation of temperature even in those which terminated favourably. Amongst the cases of anæmia it was noticed that native females seem to be able to live with a condition of the blood (absence of corpuscles and watery) far worse than that which seems to be compatible with life in Europeans, and also that the symptoms of severe anæmia seem much less marked in natives than in Europeans. The number of applicants for admission to the training school for nurses at the General Hospital fell from 94 in 1896 to 56 in 1898, but the type of candidates and the degree of their preliminary qualifications have improved. No "paying probationers" have joined the classes as yet, because accommodation could not be provided for them. Diplomas and certificates to twenty European and Eurasian midwives, and six to natives were presented during the year. At the Rajah Sir Ramaswami Mudelliar's Lying-in Hospital seven pupils who had been trained passed out in 1898, and were sent to their respective stations. The total number of operations performed in the fourteen hospitals and dispensaries of the Presidency Town of Madras during 1898 was considerably less than in the preceding years, but there was a steady increase in the number of cures, and the decrease in the percentage of deaths is very satisfactory. The total of expenditure on the medical institutions was Rs.4,11,055, which was considerably less than in the previous year owing to the smaller number of patients, cheaper rates of supply, and less expenditure upon European medicines. The subscriptions to the Victoria Caste and Gosha Hospital increased to the extent of Rs.10,143, and there was a most gratifying addition from the fees of paying patients.

